# Beyond dopamine: KarXT and emerging mechanism-based therapies in schizophrenia

**DOI:** 10.3389/fpsyt.2026.1828831

**Published:** 2026-09-23

**Authors:** Izabela Żuraw, Krystian Urbański, Patryk Kogut, Patrycja Pańczyszyn-Trzewik, Magdalena Sowa-Kućma

**Affiliations:** 1 Students Science Club “NEURON“, Faculty of Medicine, Collegium Medicum, University of Rzeszów, Rzeszów, Poland; 2 Department of Human Physiology, Faculty of Medicine, Collegium Medicum, University of Rzeszów, Rzeszów, Poland; 3 Centre for Innovative Research in Medical and Natural Sciences, Faculty of Medicine, Collegium Medicum, University of Rzeszów, Rzeszów, Poland

**Keywords:** KarXT, muscarinic receptor modulation, non-dopaminergic antipsychotic therapy, schizophrenia, trospium chloride, xanomeline

## Abstract

KarXT (marketed as Cobenfy™), a fixed-dose combination of xanomeline and trospium chloride, represents the first new mechanism of action of an antipsychotic used for adult schizophrenia approved by the US Food and Drug Administration (FDA) in over 30 years. While mainstream drugs (e.g. haloperidol, chlorpromazine, risperidone, olanzapine, quetiapine, aripiprazole) mainly antagonize dopamine D_2_ or serotonin 5-HT_2A_ receptors, KarXT acts through transient activation of muscarinic M1 and M4 receptors. M4 stimulation influences the functioning of mesolimbic dopaminergic pathways, providing an indirect means of regulating dopamine release that bypasses D2 blockade. Trospium, a peripherally acting muscarinic receptor antagonist, was used to reduce anticholinergic adverse effects (AEs). It does not cross the blood–brain barrier and therefore minimizes central adverse events. In a series of randomized placebo-controlled phase 2 and 3 studies, the KarXT significantly and clinically meaningfully reduced Positive and Negative Syndrome Scale (PANSS) total scores. The effect sizes were moderate to large with few extrapyramidal symptoms (EPS), minimal metabolic disturbances and no significant prolactin level increases. In this narrative review, we discuss in detail the pharmacological mechanism, basis of preclinical development, and clinical outcomes of KarXT. We also contextualize this drug within the development of a new wave of non-D2 antipsychotics, highlighting cholinergic dysfunction as one important mechanistic pathway within the broader multidimensional neurobiology of schizophrenia.

## 1 Introduction

Schizophrenia is a persistent and disabling mental disorder affecting approximately 23 million people worldwide (approximately 1 in 345 individuals). It typically emerges in late adolescence or early adulthood (1, 2). The disorder carries a substantial clinical and societal burden, including widespread cognitive deficits, functional impairment, and a significantly increased risk of suicide. Previous studies have estimated that approximately 9–13% of individuals with schizophrenia die by suicide during the course of their illness, although more recent meta-analyses suggest somewhat lower estimates ranging from 5% to 7% (3, 4).

Clinical symptoms of schizophrenia typically fall into three broad categories: positive symptoms (hallucinations, delusions, and disorganized thinking or speech), negative symptoms (apathy, social withdrawal, lack of pleasure, and reduced emotional expression), and cognitive impairments (deficits in working memory, attention, executive function, learning, and flexible thinking) - now recognized as central to the disorder (5, 6). It is worth noting that these cognitive problems are closely linked to long-term functional outcomes and overall quality of life for individuals with schizophrenia (5, 6).

Early biological theories related to the development of schizophrenia focused primarily on dopamine dysregulation. In particular, increased dopaminergic activity in the mesolimbic pathway was suggested to be a key driver of positive psychotic symptoms (7). However, newer models indicate that dopaminergic abnormalities may be a consequence of more profound problems related to dysfunctional neural circuits. Therefore, problems with glutamate signaling, dysfunction of GABAergic interneurons, and disturbances in cholinergic systems have been proposed as interacting factors that help explain the complex and variable clinical picture of schizophrenia (5, 8–10).

Since the introduction of chlorpromazine (a phenothiazine, first-generation antipsychotics, or FGAs), pharmacotherapy for schizophrenia has largely been centered on dopamine D_2_ receptor blockade. FGAs are potent D_2_ receptor antagonists but are associated with numerous extrapyramidal adverse effects, including acute dystonia, parkinsonism, akathisia, and tardive dyskinesia. Second-generation antipsychotics (SGAs), while retaining D_2_ antagonism, also target serotonin 5-HT_2_A receptors, generally reducing motor adverse effects but introducing other clinically significant concerns such as weight gain, dyslipidemia, and insulin resistance (11–14).

An important exception to the traditional dopamine-centered paradigm has long been clozapine, which remains the gold standard for treatment-resistant schizophrenia. Despite relatively modest D_2_ receptor occupancy, clozapine demonstrates superior efficacy through a complex pharmacological profile involving serotonergic, cholinergic, glutamatergic, and other neurotransmitter systems (8, 15, 16). Its clinical success provided early evidence that effective antipsychotic treatment may not depend solely on sustained high-level D_2_ receptor blockade.

Building on this broader pharmacological perspective, newer medications classified as third-generation antipsychotics (TGAs), such as aripiprazole and cariprazine, act as partial agonists at dopamine D_2_ and related receptors rather than as complete antagonists. By stabilizing rather than fully suppressing dopaminergic signaling, these agents may offer improved tolerability and, in some cases, benefits for negative symptoms. Cariprazine, for example, exhibits preferential affinity for D_3_ receptors, a feature proposed to contribute to its efficacy in patients with predominant negative symptoms (17, 18).

More recently, multimodal agents such as lumateperone have further expanded this concept. Lumateperone combines relatively low D_2_ receptor occupancy with serotonergic and glutamatergic modulation, achieving antipsychotic efficacy through a broader receptor profile rather than reliance on sustained high-level dopamine blockade (19). Nevertheless, despite important advances in tolerability and pharmacological sophistication, currently available antipsychotics remain only partially effective for many patients. Cognitive deficits, negative symptoms, and broader functional impairment often persist despite adequate control of positive symptoms. Furthermore, approximately 30% of patients show inadequate treatment response, and medication adherence remains a substantial clinical challenge, with reported nonadherence rates ranging from 37% to 74% (5, 20, 21). These limitations have reinforced interest in therapeutic strategies that engage neurobiological mechanisms beyond those targeted by conventional dopamine-centered treatments. In this context, nearly seventy years after the introduction of chlorpromazine, the approval of KarXT (Cobenfy) marked the first successful antipsychotic therapy based primarily on central muscarinic receptor activation rather than direct D_2_ receptor modulation. Importantly, this development does not imply that schizophrenia is primarily a cholinergic disorder. Rather, it highlights muscarinic signaling as one clinically actionable pathway within a broader multidimensional model of schizophrenia pathophysiology. It combines xanomeline, a muscarinic receptor agonist acting primarily on M1 and M4 receptors, with trospium chloride, a muscarinic receptor antagonist that does not penetrate the central nervous system but helps reduce peripheral side effects (22, 23) (see **Table 1**). Importantly, M1 and M4 receptors may contribute differently to the clinical effects of muscarinic modulation: M4 signaling is thought to influence subcortical dopaminergic tone and may be more directly linked to antipsychotic efficacy, whereas M1 receptor activation has been implicated in cortical plasticity and cognitive circuitry, with possible relevance to negative symptom domains. Mechanistically, M4 receptor activation may indirectly regulate mesolimbic dopamine release through modulation of striatal circuitry, thereby influencing psychotic symptom expression upstream of direct D_2_ receptor blockade. In contrast, M1 receptors are highly expressed in cortical regions and are closely linked to interneuron signaling, synaptic plasticity, and working memory networks within the prefrontal cortex. This distinction raises the possibility that different symptom dimensions of schizophrenia may map onto partially distinct receptor-level mechanisms, with M4-mediated pathways potentially contributing more directly to antipsychotic efficacy and M1-mediated signaling potentially more relevant to cognition and negative symptoms. Such mechanistic differentiation may have important implications for future subtype-selective muscarinic drug development and biomarker-guided patient stratification. Xanomeline was originally investigated in the 1990s as a muscarinic receptor agonist for the treatment of cognitive impairment and behavioral symptoms in neuropsychiatric conditions, particularly Alzheimer’s disease (35, 36). Subsequent clinical observations suggested that muscarinic receptor stimulation might also exert antipsychotic effects. Importantly, the seminal study by Shekhar et al. (37) demonstrated that xanomeline improved psychotic symptoms in patients with schizophrenia, providing early clinical support for muscarinic modulation as a potential antipsychotic strategy. However, despite these promising findings, xanomeline was associated with substantial peripheral cholinergic adverse effects, including nausea, vomiting, diarrhea, sweating, and hypersalivation, which limited its clinical utility (38). To address these tolerability limitations, the fixed-dose combination of xanomeline and trospium chloride (KarXT) was developed with the aim of reducing peripheral cholinergic adverse effects while preserving centrally mediated muscarinic activity.

**Table 1:** Symptom domains of schizophrenia: neurobiological mechanisms and pharmacological targets beyond the dopamine-centric model^#^.

Domain	Clinical features	Neurobiological mechanisms	Representative evidence
Positive symptoms	• hallucinations • delusions • thought disorder • disorganized behavior	• hyperdopaminergia in the mesolimbic pathway • dysregulation of glutamate-dopamine interactions	• dopamine hypothesis supported by clinical antipsychotic efficacy targeting D2 receptors • glutamatergic models suggest NMDA hypofunction contributes to dopaminergic dysregulation
Negative symptoms	• apathy • social withdrawal • reduced motivation • diminished affect	• hypodopaminergia in prefrontal cortex • glutamatergic hypofunction • disrupted cholinergic/GABAergic interneurons	• negative symptoms linked to prefrontal deficits • conventional antipsychotics show limited efficacy
Cognitive deficits	• impaired working memory • attention • executive function • cognitive flexibility	• Impaired NMDA glutamate receptor function • cortical interneuron dysfunction • possible cholinergic contribution	• cognitive deficits predict functional outcome, rarely improved by D2 blockers alone
Traditional antipsychotics (FGAs/SGAs)	• improve positive symptoms but limited on negative/cognitive	• dopamine D2 receptor blockade (plus 5-HT2A modulation in SGAs)	• efficacy on positive symptoms established • common extrapyramidal and metabolic side effects noted in literature
Novel cholinergic approach (KarXT/Cobenfy)	• reduction in both positive and negative symptoms • possible cognitive benefits (preliminary; requires dedicated prospective confirmation)	• muscarinic M1/M4 agonism modulating cholinergic circuits → indirect dopaminergic/glutamatergic effects	• KarXT significantly reduced PANSS total and subscale scores in RCTs • improvements in negative and positive symptoms

**^#^**[Based on: 5, 8, 16, 21, 22, 24–34].

D2, Dopamine D2 receptor, 5-HT2A, Serotonin 2A receptor, NMDA, N-methyl-D-aspartate receptor, M1/M4, Muscarinic acetylcholine receptor subtypes 1 and 4, PANSS, Positive and Negative Syndrome Scale, FGA, First-Generation Antipsychotic, SGA, Second-Generation Antipsychotic, EPS, Extrapyramidal Symptoms, RCT, Randomized Controlled Trial, HPA axis, Hypothalamic-Pituitary-Adrenalaxis.

The development of KarXT and related muscarinic compounds reflects a broader shift toward receptor- and circuit-informed therapeutic strategies that attempt to engage distinct dimensions of schizophrenia biology beyond exclusive D_2_ receptor antagonism. Importantly, this shift should be understood not as a rejection of dopamine biology, but as an effort to move beyond exclusive reliance on D_2_ receptor modulation toward more mechanistically diversified treatment strategies. This review examines the role of muscarinic modulation as one important component within the broader neurobiological framework of schizophrenia, which includes interacting dopaminergic, glutamatergic, GABAergic, developmental, and network-level abnormalities. Unlike previous reviews focused primarily on KarXT, the present article aims to position muscarinic therapy within a broader translational framework, emphasizing mechanistic interpretation, patient stratification, and future therapeutic positioning. Accordingly, this review critically examines the cholinergic hypothesis of psychosis, discusses the pharmacology of xanomeline and trospium chloride, and evaluates the available preclinical and clinical evidence supporting the role of KarXT in schizophrenia treatment within the wider context of emerging non-dopaminergic therapies. The novelty of this review lies not in re-summarizing KarXT trials, but in critically examining how muscarinic modulation reshapes current therapeutic thinking and informs future biologically stratified treatment models.

## 2 Methodology

This manuscript was designed as a narrative review aimed at critically synthesizing and contextualizing current evidence regarding KarXT and other emerging non-dopaminergic strategies in schizophrenia treatment. Rather than conducting a formal systematic review, we performed a targeted literature search to identify key preclinical studies, pivotal clinical trials, regulatory reports, and high-impact review articles relevant to the topic.

Electronic databases including PubMed/MEDLINE, Scopus, Embase, and Web of Science were consulted, alongside ClinicalTrials.gov and selected reference lists from key publications. Literature published between 1990 and March 2026 was considered to capture both foundational work on muscarinic receptor pharmacology and recent developments related to xanomeline–trospium (KarXT).

Priority was given to peer-reviewed primary studies, pivotal randomized controlled trials, meta-analyses, and major translational papers. The purpose of this review was interpretive and conceptual rather than exhaustive, with emphasis placed on mechanistic understanding, clinical relevance, and future therapeutic implications.

## 3 Cholinergic hypothesis of psychosis

Acetylcholine is a key neurotransmitter that influences synaptic plasticity, learning, attention, and memory by acting on two major classes of receptors: nicotinic (nAChR) and muscarinic (mAChR) (33, 39). The mAChR family consists of five subtypes (M1–M5), encoded by the *CHRM1–CHRM5* genes, which activate diverse intracellular signaling pathways. The M1, M3, and M5 subtypes are primarily Gq/11-coupled receptors that stimulate phospholipase C, exerting their effect by increasing intracellular calcium concentration. The remaining two subtypes (M2 and M4) are Gi/O-coupled receptors that act primarily inhibitory, lowering cAMP levels by inhibiting adenylate cyclase activity within the cell (39, 40). In the central nervous system, M1 receptors are abundant in the cerebral cortex and hippocampus, playing a key role in cognitive processes and synaptic plasticity, while M4 receptors are found primarily in the striatum and play a crucial role in modulating dopaminergic neurotransmission and motor control (29, 40).

Postmortem and *in vivo* neuroimaging studies consistently demonstrate a reduction in mAChR subtypes (particularly M1 and M4 subtypes) in key anterior and mid-posterior brain regions associated with the development of schizophrenia, particularly the striatum, hippocampus, and frontocingulate cortex (29, 40, 41). This provides further evidence for a breakdown in cholinergic modulation in schizophrenia and an increase in dopaminergic dysregulation mediated by cholinergic dysfunction, which may impact both clinical and cognitive aspects of the disorder (29, 40, 41). M4 receptor activation inhibits dopamine release in striatal circuits by directly reducing presynaptic cholinergic activity, leading to decreased dopaminergic transmission via the indirect pathway (32). This contrasts with classical antipsychotics, which act by directly blocking postsynaptic D2 dopamine receptors, promoting a “dopamine-sparing” model in which cholinergic modulation regulates dopaminergic tone without direct D2 receptor antagonism. By suppressing excessive mesolimbic dopamine release (via activation of M4 receptors), muscarinic modulation may alleviate positive psychotic symptoms while potentially avoiding the EPS associated with direct D2 receptor blockade (29, 40). Most medications currently used to treat schizophrenia, unlike first-generation antipsychotics (FGAs; haloperidol, chlorpromazine, fluphenazine), are second-generation antipsychotics (SGAs; risperidone, olanzapine, quetiapine, clozapine, ziprasidone), which antagonize both dopamine and serotonin receptors. Although these medications effectively alleviate positive symptoms, they are ineffective in treating negative and cognitive symptoms and are considered poorly tolerated due to their high number of side effects. Antipsychotic resistance occurs in approximately one-third of this population for reasons that remain incompletely understood, posing a significant therapeutic challenge (42). Furthermore, nonadherence to antipsychotic treatment remains a significant clinical problem; rates vary widely (often around 40–60% in many cohorts) depending on study methods and patient populations and are also influenced by factors such as lack of insight and medication side effects (43, 44). Antipsychotic medications also carry a risk of cardiometabolic adverse effects, such as weight gain, dyslipidemia, insulin resistance, and an increased risk of metabolic syndrome. Longer exposure periods (>90 days) have been associated with an increased risk of metabolic syndrome events (e.g., hypertension, type 2 diabetes) in large population-based databases, although direct associations with major cardiovascular events (e.g., myocardial infarction) have been inconsistent across age groups and populations (45). These metabolic side effects are important factors explaining the increased cardiovascular morbidity and mortality in individuals with serious mental illnesses, including schizoaffective disorder (45, 46). Peripheral side effects and metabolic burden also contribute to poor medication adherence and poorer long-term treatment outcomes (43, 44). Among motor side effects of antipsychotics, extrapyramidal symptoms (EPS) such as parkinsonism, akinesia, akathisia and tardive dyskinesia are predominantly associated with D2 receptor blockade in the basal ganglia (47). FGAs tend to cause EPS more frequently due to robust D2 antagonism, whereas SGAs also antagonize 5-HT2A receptors - a profile that may mitigate EPS but is linked to weight gain and metabolic syndrome (48) (see also **Table 1**).

## 4 Xanomeline and trospium: pharmacological synergy

### 4.1 Xanomeline

Xanomeline [3-(3-hexyloxy-1,2,5-thiadiazol-4-yl)-1,2,5,6-tetrahydro-1-methylpyridine –TZTP] (the structural formula is shown in **Figure 1A**) with a molar mass of 281.42 g/mol (49) is a central nervous system (CNS)-penetrating M1 and M4 receptor agonist, confirming a different mechanism of action from most drugs used (or tested) in the treatment of schizophrenia (24, 30). Preclinical data indicate a favorable profile of action in the CNS. However, concomitant peripheral cholinergic activation led to gastrointestinal intolerance in early studies (37, 50). This limitation led to the development of KarXT, in which xanomeline was coadministered with trospium chloride to reduce peripheral side effects (30, 51).

**Figure 1:**
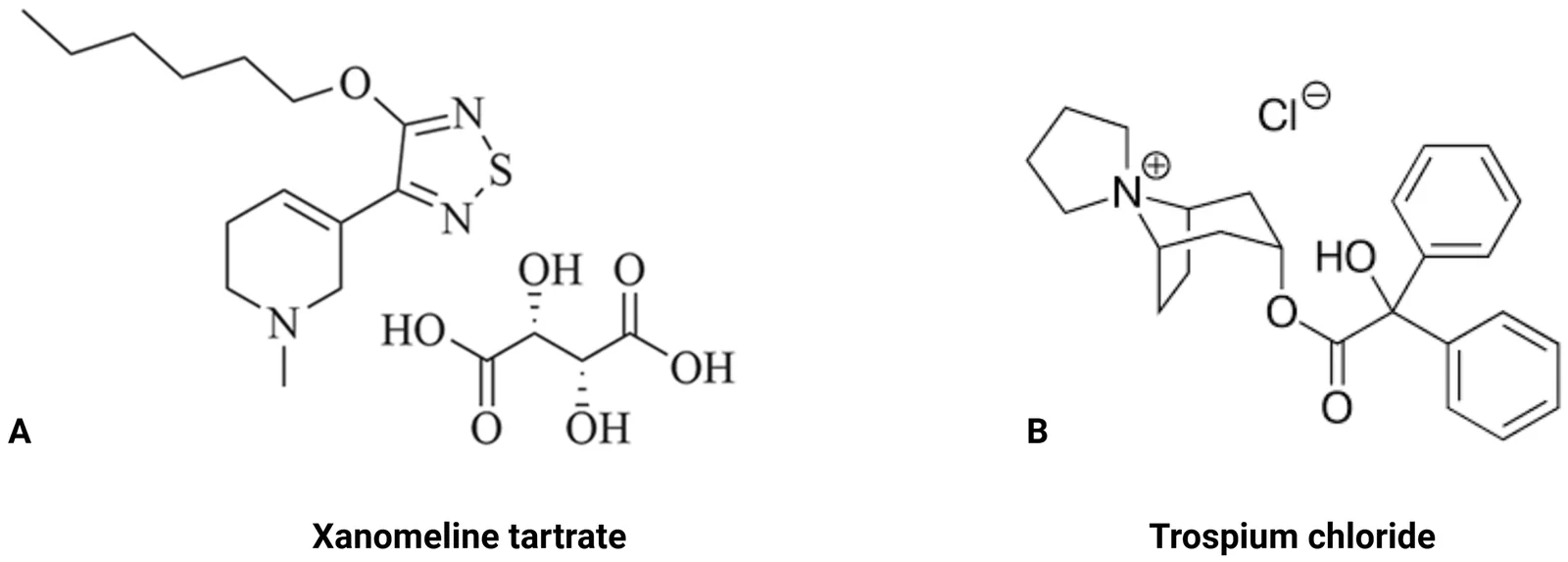
The chemical structure of xanomeline tartrate **(A)** and trospium chloride **(B)**. (Created in BioRender).

#### 4.1.1 Pharmacokinetics

Preclinical studies in rats and monkeys demonstrated rapid absorption of xanomeline following oral administration. In monkeys, brain concentrations were 10–20 times higher than in plasma, with peak concentrations occurring in the cerebral cortex and striatum (consistent with the high density of M1/M4 receptors), while concentrations in other brain regions were approximately half of the peak concentrations (52).

Xanomeline is metabolized primarily in the liver by the enzymes CYP2D6, CYP2B6, CYP1A2, CYP2C9, and CYP2C19 (49). Human studies have shown maximum plasma concentrations (Tmax) of 2–2.5 hours after oral administration, with a mean Cmax of 8.95 ng/ml for a 100 mg dose and 13.81 ng/ml for a 150 mg dose (49). According to the FDA drug label, the maximum absorption time is 2 hours, the plasma half-life is 5 hours, and the renal clearance is 0.085 l/h. The drug is excreted primarily in urine (78%) and feces (12%) (51).

#### 4.1.2 Pharmacodynamics

Xanomeline is a muscarinic receptor agonist with affinity for all five subtypes (10, 12, 17, 7, and 22 nM, respectively, for M1–M5) (51). Nevertheless, the strongest agonistic effects are observed for M1 and M4 receptors, which are abundant in the cerebral cortex, hippocampus and striatum (24, 49). Furthermore, *in vitro* studies indicate low affinity of xanomeline for D2, 5-HT3, α1, α2, β1, β2, opioid, glycine, and GABA receptors, confirming its selectivity for cholinergic pathways (49, 51). Preclinical studies have revealed a unique muscarinic receptor-based activity profile that highlights schizophrenia-specific clinical mechanisms that xanomeline may potentially regulate. *In vivo* studies demonstrate that xanomeline reduces the activity of A10 dopaminergic neurons in the ventral tegmental area, reducing dopamine release in the mesolimbic system. The involvement of this pathway has been strongly implicated in the pathophysiology of positive psychotic symptoms (53). Importantly, xanomeline does not induce catalepsy in animal models, indicating a low propensity for EPS typically associated with direct dopamine D_2_ receptor antagonism (53). Neurobiological mapping studies have shown regionally selective activation patterns, including c-Fos induction in the prefrontal cortex with minimal activation in the striatum, suggesting preferential engagement of cortical regulatory networks rather than motor circuits (53). Importantly, M1 and M4 receptor activation may contribute differently to KarXT’s clinical effects. M4 receptors, enriched in subcortical regions, modulate dopaminergic signaling upstream of D_2_ pathways and may primarily mediate antipsychotic effects on positive symptoms. In contrast, M1 receptors are highly expressed in cortical regions, where they regulate synaptic plasticity and working memory circuits, potentially contributing to cognitive and negative symptom domains. The relative contribution of these receptor subtypes remains an important unresolved question and may have implications for future patient stratification and development of more selective muscarinic agents.

In behavioral studies, xanomeline reverses deficits induced by amphetamine and MK-801 (a potent noncompetitive N-methyl-D-aspartate-NMDA receptor antagonist in the central nervous system), suggesting it may be effective on positive, negative, and cognitive symptoms (54). It also reduces psychotic behavior in nonhuman primates without causing motor side effects, increasing its potential clinical relevance (52). Xanomeline readily crosses the blood-brain barrier, allowing it to influence central cholinergic pathways and thus modulate dopamine release in the midbrain and striatal regions. This dual action contributes to its effects on both positive and negative symptoms of schizophrenia (30, 55). Importantly, concurrent activation of peripheral muscarinic receptors can cause dose-dependent gastrointestinal problems such as diarrhea, vomiting, and hypersalivation. These side effects have historically limited its use in clinical settings (24, 37). The successful solution was to combine xanomeline with trospium chloride (a muscarinic receptor antagonist that does not penetrate the brain), which reduces these peripheral side effects without suppressing the activity of central muscarinic receptors, thus enabling targeted treatment of the central nervous system while improving treatment tolerability (30, 51).

### 4.2 Trospium chloride

Trospium chloride (its structural formula is shown in **Figure 1B**) is a quaternary ammonium compound chemically classified as a nortropane derivative, structurally similar to atropine-like compounds. Its molar mass is approximately 392.5 g/mol (56) and it acts as a non-selective competitive antagonist of mAChRs. It exhibits activity primarily against peripheral subtypes: M1, M2, and M3, with slightly lower affinity for M4 and M5 receptors (56, 57). Trospium does not bind significantly to nAChRs and is highly hydrophilic, which makes it almost impossible to cross the blood-brain barrier (56, 57). The firstFDA approval of trospium in the United States came in 2004 and was for overactive bladder, a condition in which the main symptoms are urinary urgency, frequency, and incontinence (57). As a peripheral muscarinic receptor antagonist, trospium plays a key role in reducing AEs when combined with xanomeline in a fixed-dose formulation known as KarXT for the treatment of schizophrenia (57).

#### 4.2.1 Pharmacokinetics

After oral administration, trospium chloride exhibits poor and incomplete absorption from the gastrointestinal tract (mean absolute bioavailability in humans is less than 10%) (57, 58). Maximum plasma concentrations typically occur approximately 5 to 6 hours after administration (Tmax) and are approximately 4 to 7 ng/ml (Cmax) after standard immediate-release doses (20 mg) (56, 58). Consuming food, especially high-fat food, can drastically reduce drug absorption, sometimes reducing AUC and Cmax by as much as 70 to 80%. For this reason, it is recommended to take trospium on an empty stomach or at least one hour before a meal (51, 58). Trospium has a large apparent volume of distribution of approximately 350–800 liters, suggesting extensive dispersion into peripheral tissues (58). Plasma protein binding is moderate, approximately 50%, and the compound undergoes minimal hepatic metabolism. Its metabolism occurs primarily via non-CYP450 pathways, likely involving ester hydrolysis and subsequent glucuronidation. This metabolic profile means that few drug interactions are expected (51, 57, 58). Importantly, trospium is not significantly metabolized by cytochrome P450 enzymes, distinguishing it from many other anticholinergic medications (51, 57). The half-life of trospium is typically about 10 to 20 hours, although some formulations and patient populations report longer elimination periods (51, 58). Renal clearance is particularly significant (approximately 29 liters/hour), with urinary excretion playing a dominant role in drug elimination, accounting for approximately 70% of total clearance. The parent drug is excreted primarily unchanged in both urine and feces. The exact proportions vary by formulation, with some sources suggesting approximately 85% is excreted in feces and only 5–10% in urine (51, 57, 58). Since trospium clearance closely correlates with renal function, in patients with significantly impaired renal function, i.e. creatinine clearance below 30 ml/min, dosage adjustment is recommended to prevent drug accumulation (58).

#### 4.2.2 Pharmacodynamics

Trospium chloride acts as a competitive antagonist of the muscarinic receptor mAchR, blocking the effects of acetylcholine on smooth muscle and secretory tissues in the autonomic nervous system (56, 57). It binds with high affinity to the M1, M2, and M3 receptor subtypes, thereby inhibiting cholinergic signals responsible for detrusor contractions, glandular secretions, and smooth muscle tone in the lower urinary tract (56, 57). This cholinergic blockade reduces involuntary bladder contractions and increases maximum bladder capacity measured by cystometry. Furthermore, it reduces urinary urgency and frequency, leading to improved urinary continence in patients with overactive bladder (51, 57). Trospium’s competitive antagonism also explains its antispasmodic effect on smooth muscle, reducing gastrointestinal muscle tone (51, 57). Importantly, trospium does not readily cross the blood-brain barrier, resulting in minimal AEs in the central nervous system. This contrasts with the AEs of tertiary amines such as oxybutynin, which often cause cognitive impairment (57, 58). This peripheral selectivity is particularly useful in formulations such as KarXT, where trospium helps reduce the peripheral AEs of xanomeline, such as gastrointestinal discomfort and hypersalivation, without interfering with the central agonist effects of xanomeline in the treatment of schizophrenia (57).

The most frequently observed side effects of trospium treatment are typical of anticholinergic medications. These include dry mouth, constipation, dyspepsia, and abdominal discomfort. These symptoms primarily reflect blockade of peripheral muscarinic receptors rather than effects on the central nervous system (56, 57).

## 5 Preclinical insights into xanomeline: antipsychotic potential and muscarinic mechanisms

Preclinical studies of xanomeline, which preferentially blocks M1 and M4 muscarinic acetylcholine receptors, have revealed a consistent profile of antipsychotic activity in several animal models. The observed effects include electrophysiological, behavioral, neurochemical, and gene expression changes consistent with antipsychotic activity.

Preliminary studies have shown that xanomeline exerts antipsychotic effects in both rats and mice in various dopamine-related models. Electrophysiological data showed that both acute and chronic xanomeline administration inhibited the activation of dopaminergic neurons in the A10 region (ventral tegmental area) but did not affect neurons in the A9 region (substantia nigra). This inhibition was blocked by scopolamine (a muscarinic receptor antagonist), suggesting that the observed effects are mediated by muscarinic receptors rather than direct dopamine receptor blockade. Behaviorally, xanomeline reversed apomorphine-induced climbing in mice and dopamine agonist-induced turning in rats with unilateral 6-OHDA lesions, effects similar to those observed with haloperidol, clozapine, and olanzapine. Importantly, xanomeline did not induce catalepsy in rodents, even at doses demonstrating antipsychotic efficacy, which is often used as an indicator of EPS (53).

Microdialysis experiments examining neurochemical effects demonstrated that xanomeline increased extracellular dopamine levels in the prefrontal cortex (PFC) and nucleus accumbens (NAc), while leaving dopamine unchanged in the striatum. Concomitantly, increased expression of early genes, such as c-fos and zif/268, was observed in the PFC and NAc, but not in the striatum, suggesting region-specific neuronal activation. This profile is somewhat similar to the response induced by atypical antipsychotics (such as clozapine or olanzapine), suggesting the involvement of neural circuits related more to antipsychotic action than to motor side effects (34).

In various behavioral tests, xanomeline reduced amphetamine-induced hyperlocomotion and reversed apomorphine- or amphetamine-induced impairments in prepulse inhibition (PPI) in rodents. These results are consistent with expectations for substances targeting positive symptoms observed in schizophrenia (59). Activation of M1/M4 muscarinic receptors also appeared to counteract experimentally induced deficits that model negative and cognitive symptoms of schizophrenia. For example, in latent inhibition tasks, xanomeline reversed the impairments induced by amphetamine, scopolamine, and MK-801, indicating that its effects extend beyond positive symptoms and involve disease-related cognitive areas (54).

Beyond the classical framework of psychosis, xanomeline has demonstrated analgesic properties in models of inflammatory and neuropathic pain, suggesting that central muscarinic signaling, particularly via M1 receptors, plays a role in supraspinal pain modulation. Blockade of these analgesic effects by muscarinic drugs such as scopolamine and pilocarpine supports the hypothesis of a muscarinic-dependent mechanism (60). Animal studies have further demonstrated xanomeline’s effects on arousal and wakefulness. In rodent models of aging, treatment improved wakefulness by reducing fragmentation and increasing arousal compared to controls. Interestingly, these effects were not observed with donepezil (an acetylcholinesterase inhibitor), suggesting that xanomeline may modulate cortical cholinergic circuits involved in attention and vigilance (50).

In non-human primates, xanomeline demonstrated pronounced antipsychotic activity: it inhibited d-amphetamine- and apomorphine-induced restlessness, stereotypies, and arousal behaviors in Cebus apella monkeys at doses that did not induce EPS, although vomiting occurred at higher doses, consistent with peripheral cholinergic activation observed in humans. Even in EPS-sensitized monkeys, xanomeline maintained antipsychotic efficacy without causing motor adverse effects at therapeutic doses ranging from 0.5 to 3 mg/kg (52).

Given these findings, it is reasonable to conclude that xanomeline’s antipsychotic effects likely result from modulation of M1/M4 muscarinic receptors, which in turn influence dopaminergic and cholinergic neurotransmission. This translates into benefits in behavior, cognition, and neurochemical activity, importantly, often without EPS. This distinguishes xanomeline from more traditional dopamine antagonists. However, its early clinical use was hampered by peripheral AEs, prompting the development of combination therapies (such as with trospium) aimed at improving tolerability.

## 6 Preclinical insights into trospium chloride: peripheral muscarinic antagonism and safety in combination with xanomeline

Trospium chloride, a quaternary ammonium muscarinic antagonist, has been characterized preclinically for its strong peripheral muscarinic receptor blockade and limited penetration into the central nervous system. While it doesn’t exhibit antipsychotic effects itself, its pharmacological profile makes it a suitable partner for xanomeline by reducing peripheral AEs without impeding central M1/M4 receptor activation.

### 6.1 Preclinical pharmacology and safety profile of trospium chloride

Ex vivo experiments using human and porcine detrusor muscle strips showed that trospium competitively inhibited acetylcholine- and carbachol-induced contractions, suggesting an action at M1–M3 muscarinic receptors in smooth muscle (57, 61). These studies confirm the ability of trospium to reduce peripheral cholinergic stimulation at nanomolar concentrations. Comparable inhibitory effects were noted in gastrointestinal smooth muscle, consistent with its known parasympatholytic activity. Studies using *in vivo* models of lower urinary tract function in rodents have shown that trospium reduces bladder contractility in a dose-dependent manner, consistent with its role as a muscarinic receptor antagonist (62). Toxicology studies conducted in rodents also revealed expected peripheral side effects, such as decreased gastrointestinal motility, mild heart rate acceleration, and mydriasis, but without evidence of CNS effects. These peripheral effects are consistent with clinical observations and define a peripheral safety profile that supports the use of trospium in combination therapies.

### 6.2 Blood-brain barrier properties and rationale for combination with xanomeline (KarXT)

Rodent experiments suggest that trospium practically does not cross the blood-brain barrier, even in aged animals. This limited penetration is attributed to its polar, quaternary ammonium structure and active clearance by transporters such as P-glycoprotein (63). Consequently, central cholinergic functions (including cognitive and motor functions) remain essentially unaffected, supporting the rationale for co-administration of trospium with centrally acting muscarinic agonists such as xanomeline.

Pharmacologically, trospium appears to selectively block peripheral muscarinic receptors activated by xanomeline, thereby reducing gastrointestinal and autonomic side effects such as nausea, vomiting, and hypersalivation. Importantly, it does not interfere with central xanomeline M1/M4 receptor activity, allowing the agonist to retain its antipsychotic and procognitive properties in preclinical models.

Studies in rodents and primates examining combinations of muscarinic agonists and antagonists have shown that peripheral blockade with quaternary amines does not reduce central c-Fos activation or behavioral responses induced by central M1/M4 receptor agonists. This supports the therapeutic approach of combining xanomeline with trospium to improve tolerability without compromising efficacy (56, 57, 61).

## 7 Clinical development of xanomeline and the xanomeline–trospium combination: from early proof-of-concept to phase 3 validation

### 7.1 Early clinical evidence for xanomeline: cognitive and antipsychotic proof-of-concept

Early clinical trials of xanomeline, primarily in patients with Alzheimer’s disease, provided preliminary evidence of antipsychotic and cognitive benefits derived from central muscarinic agonism. In a multicenter, randomized, double-blind study conducted at 17 sites in the United States and Canada, participants received placebo or xanomeline at doses of 75 mg, 150 mg, or 225 mg daily. Cognitive assessment using the Alzheimer’s Disease Assessment Scale – Cognitive Subscale (ADAS-Cog) showed dose-dependent improvements in areas such as spatial perception, orientation, language, and word retrieval, particularly at the highest dose. Behavioral symptoms (measured by the Alzheimer’s Disease Symptom Scale; ADSS) were also significantly reduced, including reductions in verbal outbursts, suspiciousness, hallucinations, agitation, and wandering, clearly indicating a dose-response relationship (36). At the same time, peripheral AEs, such as nausea, vomiting, and diarrhea, were common and significantly limited the ability to increase the dose. This highlights the common problem of managing peripheral tolerance in muscarinic agonist therapy. Nevertheless, this study provides proof of concept for the cognitive and behavioral effects stimulated by activation of M1/M4 muscarinic receptors in humans. Based on these findings, Shekhar et al. (37) conducted a four-week, double-blind, placebo-controlled pilot study examining xanomeline monotherapy in 20 patients with acute schizophrenia (PANSS >60). Dosing began at 25 mg three times daily and was increased to 75 mg based on tolerability. The study demonstrated a statistically significant reduction in the PANSS total score compared with placebo (mean reduction of 24.0 ± 21.0 points, p = 0.039). Although improvements in the positive, negative, and clinical global impression (CGI) subscales did not reach statistical significance, the results still indicated potential efficacy. Gastrointestinal side effects were generally mild to moderate, and importantly, no extrapyramidal symptoms (EPS) or cardiovascular changes were reported (37). This study highlighted both the potential of xanomeline as an antipsychotic and the ongoing challenge of managing peripheral AEs in this patient group.

### 7.2 Pharmacological strategy to improve tolerability: addition of trospium

To understand and tackle tolerability issues, Breier et al. (64) conducted a 9-day, phase 1, double-blind trial (NCT02831231) involving healthy adults (see also **Table 2**). Subjects received either xanomeline alone (75 mg three times daily, n=33) or xanomeline combined with trospium (20 mg twice daily, n=35). Co-administration of trospium led to a 46% reduction in peripheral cholinergic adverse events compared to xanomeline monotherapy. Notably, sweating decreased by 59%, vomiting by 62%, and nausea by 29%. Interestingly, trospium did not impact the pharmacokinetics of xanomeline, nor did it affect vital signs, laboratory parameters, or ECG readings (64). This suggested that peripheral anticholinergic blockade could improve tolerability without diminishing the central muscarinic effects of xanomeline. These results laid the groundwork for the development of KarXT, a combination therapy aimed at preserving central M1/M4 receptor activity while minimizing peripheral side effects.

**Table 2:** Clinical development program of xanomeline–trospium (KarXT) in schizophrenia: study design, efficacy outcomes, and safety profile^#^.

Trial	ID	Phase	N	Duration	Dosing (mg BID)	PANSS change (placebo-controlled studies: between-group difference; open-label studies: within-group change from baseline)	Cohen’s d	≥30% PANSS responders	Most common AEs	EPS / weight / metabolic
Phase 1 Tolerability Study	NCT02831231	1	68 (33 mono / 35 combo)	9 days	Xano 75 mg TID ± Trospium 20 mg BID	—	—	—	↓ Sweating 59%, ↓ Vomiting 62%, ↓ Nausea 29% (46% overall AE reduction vs mono)	None / none/ neutral
EMERGENT-1	NCT03697252	2	182	5 weeks	50/20 → 125/30	−17.4 vs −5.9	0.75	—	Nausea 17%, Constipation 17%, Dry mouth 9%, Vomiting 9%	None / none / neutral
EMERGENT-2	NCT04659161	3	252	5 weeks	Up to 125/30	−21.2 vs −11.6	0.61	—	Constipation 21%, Nausea 19%, Dyspepsia 19%, Vomiting 14%	None / none / neutral
EMERGENT-3	NCT04738123	3	256	5 weeks	Up to 125/30	−20.6 vs −12.2	0.60	—	Nausea 19%, Vomiting 16%, Dyspepsia 16%, Constipation	None / none / neutral
EMERGENT-4	NCT04659174	3 OL	156	52 weeks	50/20 → 125/30	−33.8 vs −31.3*	—	69%	Gastrointestinal (nausea, vomiting, dyspepsia), dry mouth, hypertension	None / −1.9 kg / neutral
EMERGENT-5	NCT04820309	3 OL	566	52 weeks	50/20 → 125/30	−5.5 (baseline ~66)*	—	30%	GI AEs: nausea, vomiting, dyspepsia, dry mouth, hypertension	None / −2.2 kg / neutral

*Responder rates reported in EMERGENT-4 and EMERGENT-5 reflect within-cohort outcomes and are not directly comparable to placebo-controlled response estimates.

^#^[Based on: 22, 24–26, 38, 64–69].

PANSS, stands for Positive and Negative Syndrome Scale and is a standardized tool to assess the severity of positive and negative symptoms in schizophrenia; CGI-S or Clinical Global Impression – Severity, is a clinician-rated measure of overall illness severity; AE, refers to Adverse Event, indicating any undesirable experience associated with treatment; EPS, denotes Extrapyramidal Symptoms, which include movement disorders such as tremors, rigidity, or involuntary movements that can occur with certain psychotropic medications; BID (bis in die), indicates a twice-daily dosing schedule; Cohen’s d (d), is a standardized measure of effect size; while CI (Confidence Interval), represents the statistical range within which the true effect is likely to lie. Finally; NCT, is the registration number of the clinical trial in the ClinicalTrials.gov database.

### 7.3 Clinical effectiveness studies – EMERGENT trials

The EMERGENT program represented a thorough clinical investigation of KarXT (xanomeline-trospium) for treating acute schizophrenia. It comprised phase 2 and phase 3 studies, as well as long-term open-label trials, collectively providing a substantial dataset for assessing efficacy, safety, and tolerability of this muscarinic-based antipsychotic candidate (see also **Table 2**).

#### 7.3.1 Phase 2 clinical validation: EMERGENT-1

The phase 2 EMERGENT-1 study (NCT03697252) was a 5-week, double-blind, placebo-controlled study of 182 adults aged 18–60 years diagnosed with schizophrenia (according to DSM-5 criteria) and experiencing an acute exacerbation. Participants were randomly assigned to receive KarXT, starting with 50 mg xanomeline/20 mg trospium twice daily, titrated to 125 mg/30 mg twice daily, or placebo. The study successfully met its primary endpoint, demonstrating a significant reduction in the PANSS total score for KarXT (−17.4 ± 1.8) compared with placebo (−5.9 ± 1.7). A difference of -11.6 points (95% CI -16.1 to -7.1, P < 0.001; Cohen’s d = 0.75) indicates a moderate to large effect size. Symptoms improved early, with significant changes observed as early as week 2. Secondary analyses demonstrated benefits on the positive and negative subscales of the PANSS, the Marder Five-Factor Scale, and the CGI-S, reflecting significant symptom improvement (70).

The tolerability profile of KarXT was generally favorable. Adverse events during treatment were primarily mild to moderate gastrointestinal complaints, such as nausea (17%), constipation (17%), dry mouth (9%), and vomiting (9%). Importantly, no extrapyramidal symptoms, weight gain, or significant metabolic disturbances were reported, distinguishing this treatment from many existing antipsychotics (68).

#### 7.3.2 Phase 3 confirmation: EMERGENT-2 and EMERGENT-3

Subsequent Phase 3 studies, EMERGENT-2 (n=252) and EMERGENT-3 (n=256), extended these results by examining adults with acute schizophrenia for 5 weeks, using flexible dosing up to a maximum of 125 mg xanomeline/30 mg trospium twice daily. Both studies confirmed the efficacy observed in EMERGENT-1.

In EMERGENT-2, treatment with KarXT resulted in a reduction in the PANSS total score of -21.2 ± 1.7 points compared with -11.6 ± 1.6 points for placebo. The observed difference of -9.6 points (95% CI -13.9 to -5.2; Cohen’s d = 0.61) further supports the clinical significance of these results (25). Similarly, the EMERGENT-3 trial demonstrated a reduction of -20.6 ± 1.6 compared with -12.2 ± 1.6 for placebo (difference -8.4; 95% CI -12.4 to -4.3; Cohen’s d = 0.60). Secondary endpoints in both trials demonstrated consistent improvement in positive, negative, and disorganized symptoms. The most frequently observed adverse events were mild to moderate cholinergic symptoms: nausea (16–19%), vomiting (14–16%), constipation (16–21%), and dyspepsia (16–19%). Again, no extrapyramidal symptoms, weight gain, or metabolic changes were observed, underscoring the good tolerability of KarXT (22, 26). Preliminary *post-hoc* analyses from the EMERGENT program suggest possible cognitive benefit in patients with baseline impairment; however, these findings remain exploratory, and dedicated prospective studies using standardized instruments such as the MATRICS Consensus Cognitive Battery are still needed before definitive conclusions can be drawn.

#### 7.3.3 Long-term safety and functional outcomes: EMERGENT-4 and EMERGENT-5

The EMERGENT-4 and EMERGENT-5 studies were designed as 52-week, open-label outpatient extensions to evaluate the long-term safety, tolerability, and durability of response associated with KarXT treatment (65, 66).

EMERGENT-4 (n = 156) enrolled patients who had previously completed EMERGENT-2 or EMERGENT-3. PANSS total scores decreased by −33.8 points among patients continuing KarXT and by −31.3 points among those switching from placebo, with approximately 69% of participants achieving at least a 30% reduction in PANSS scores. The most frequently reported adverse events were gastrointestinal in nature, while mean body weight decreased slightly (−1.9 ± 4.7 kg). Importantly, no new safety signals emerged during long-term follow-up, and rates of extrapyramidal symptoms, prolactin-related adverse effects, and metabolic disturbances remained low (66).

EMERGENT-5 (n = 566) extended these observations to a broader outpatient population with stable schizophrenia previously treated with other antipsychotic medications. Mean PANSS scores improved by approximately 33 points over 52 weeks, while improvements in CGI-S scores and PANSS symptom domains were generally consistent with findings from earlier studies. Weight remained stable or decreased modestly (−2.2 ± 6.3 kg), and metabolic parameters, including cholesterol, triglycerides, and HbA1c, showed little change over time. Patient-reported measures also suggested sustained improvements in quality of life and treatment satisfaction throughout follow-up (65).

Taken together, the EMERGENT-4 and EMERGENT-5 studies provide supportive evidence that the efficacy observed in the acute EMERGENT trials may be maintained over longer treatment periods while preserving a favorable metabolic and extrapyramidal safety profile (65, 66). However, given their open-label design and absence of active comparators, conclusions regarding long-term comparative effectiveness remain preliminary.

#### 7.3.4 Unresolved questions and current limitations

Despite the encouraging findings generated by the EMERGENT program, several important questions remain regarding the long-term clinical role of KarXT in schizophrenia treatment.

First, no randomized active-comparator studies have yet established whether KarXT provides advantages over established second-generation antipsychotics with respect to efficacy, tolerability, treatment discontinuation, or functional outcomes. Consequently, its relative position within current treatment algorithms remains uncertain.

Second, although short-term antipsychotic efficacy has been demonstrated consistently across EMERGENT-1 through EMERGENT-3, robust controlled evidence for relapse prevention, maintenance treatment, hospitalization reduction, suicidality, aggression, and long-term functional recovery remains limited. Existing long-term studies primarily provide open-label safety and durability-of-response data rather than definitive maintenance efficacy evidence (22, 25, 26, 65, 66, 71).

Third, the potential role of KarXT in treatment-resistant schizophrenia remains unclear. Given the unique efficacy profile of clozapine and the biological heterogeneity of schizophrenia, it is uncertain whether muscarinic modulation may benefit patients with true dopamine-resistant illness or instead identify a partially distinct treatment-responsive subgroup.

Similarly, although exploratory analyses have suggested possible benefits in cognition and negative symptoms, these findings remain preliminary and require confirmation in prospective studies specifically designed to assess domain-specific outcomes (27).

Finally, identifying biomarkers and clinical characteristics associated with response to muscarinic modulation remains a major research priority and may ultimately help define which patients derive the greatest benefit from KarXT.

### 7.4 Practical prescribing considerations

Beyond efficacy demonstrated in clinical trials, the future clinical utility of KarXT will likely depend on how its unique efficacy and tolerability profile translates into routine clinical practice. Its non-dopaminergic mechanism and favorable extrapyramidal and metabolic profile may make KarXT particularly attractive for patients who experience extrapyramidal symptoms, hyperprolactinemia, or metabolic complications during conventional antipsychotic treatment (65, 66, 68).

At the same time, KarXT introduces a distinct tolerability profile that differs from traditional dopamine-based therapies. The most commonly reported adverse events across the EMERGENT program have included nausea, constipation, dyspepsia, vomiting, and dry mouth, which were generally mild to moderate in severity and often transient (22, 25, 26, 65, 66, 68). Consequently, KarXT may shift rather than eliminate treatment-related tolerability burdens, requiring individualized assessment of benefit-risk tradeoffs.

Current prescribing decisions must also account for practical factors such as twice-daily dosing, which may influence adherence in some patients, particularly those with chronic illness burden or cognitive impairment.

From a clinical perspective, KarXT should currently be viewed as a complementary addition to existing antipsychotic options rather than a replacement for established therapies. Patients who poorly tolerate dopamine-based agents may represent an especially relevant population for its use, although broader experience and longer-term evidence will be needed to better define its optimal place in treatment algorithms.

## 8 KarXT in the landscape of non-D_2_ antipsychotic therapies

For nearly 70 years, the development of antipsychotic therapies has focused primarily on compounds acting through full or partial antagonism of the dopamine D_2_ receptor. This dopamine-based approach remains the mainstay of schizophrenia treatment, effectively reducing positive symptoms such as hallucinations and delusions. However, dopamine receptor blockade carries a number of significant side effects, including extrapyramidal symptoms, elevated prolactin levels, and metabolic disturbances, which can be particularly severe with certain SGAs. Furthermore, drugs targeting D_2_ receptors typically have limited or inconsistent efficacy in combating negative and cognitive symptoms, which are key factors contributing to long-term disability and reduced quality of life in patients with schizophrenia (21). Due to these limitations, alternative pharmacological approaches focusing on non-dopaminergic mechanisms are gaining increasing interest. Candidates include not only muscarinic receptor agonists (like KarXT) but also trace amine-associated receptor 1 (TAAR1) agonists, serotonergic modulators, and glutamatergic agents (**Figure 2**). Although some early studies of these compounds showed promising results, most have encountered challenges, such as inconsistent efficacy, tolerability issues, or failure to replicate benefits in larger, controlled trials (24, 72). **Table 3** provides a comparative overview of these novel therapeutic strategies.

**Figure 2:**
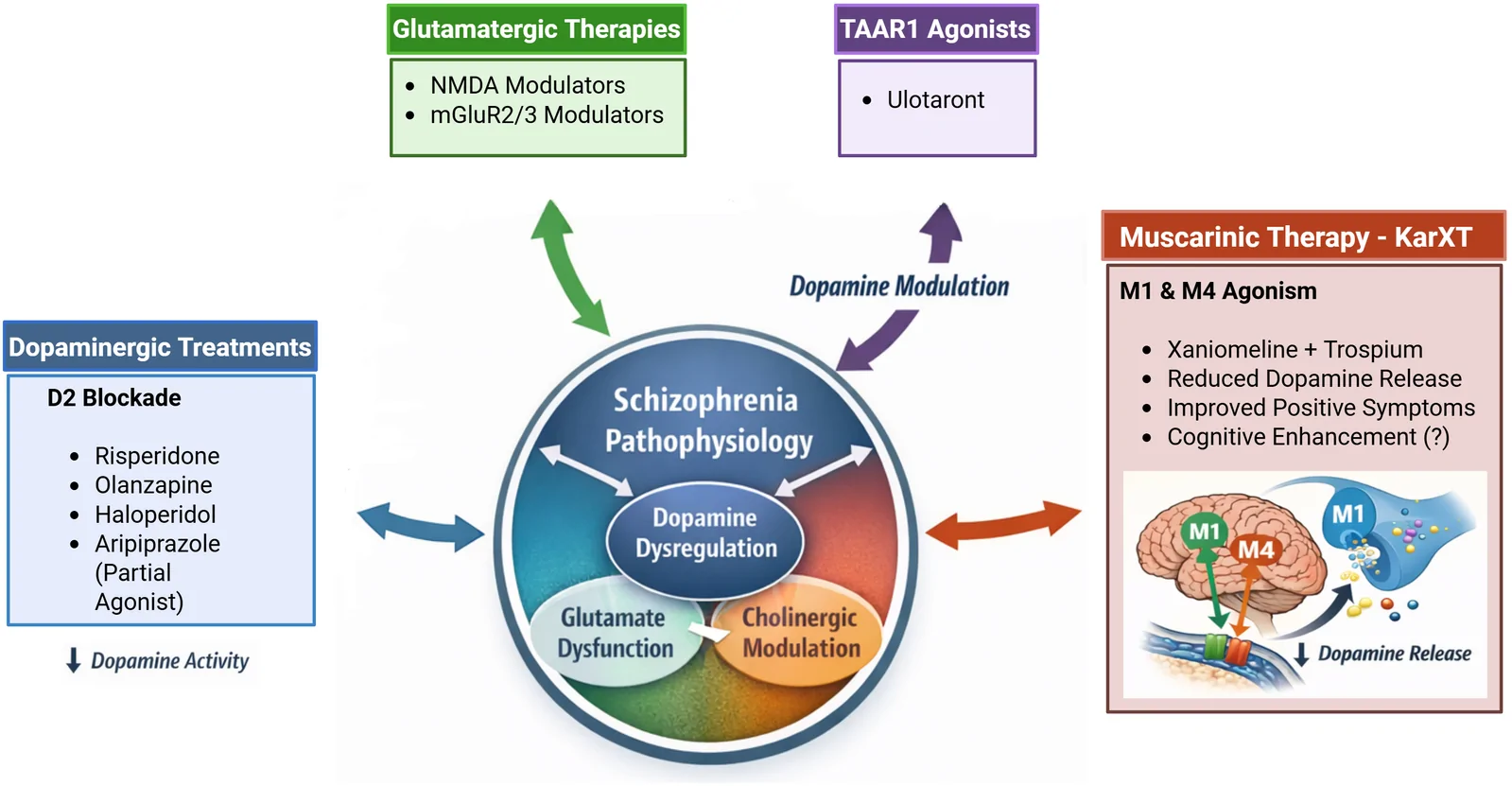
Current and emerging pharmacological targets in schizophrenia with positioning of KarXT. (Created in BioRender).

**Table 3:** Emerging alternatives and complementary strategies beyond traditional D2 antagonism^#^.

Drug / strategy	Mechanism of action	Key clinical trials	Main efficacy findings	Safety profile
KarXT (xanomeline–trospium)	Central M1/M4 muscarinic receptor agonism (xanomeline) combined with peripheral muscarinic antagonism (trospium) to limit cholinergic adverse effects	EMERGENT-1 (NCT03697252), EMERGENT-2 (NCT04659161), EMERGENT-3 (NCT04738123), EMERGENT-5 (NCT04820309)	Significant reduction in PANSS total score vs placebo (LS mean difference ~ −9 to −10; Cohen’s d ≈0.60–0.75). Improvements observed across positive and negative symptom domains	Mostly gastrointestinal AEs (constipation, nausea, dyspepsia). No clinically meaningful EPS, weight gain, or metabolic abnormalities
Ulotaront (SEP-363856)	TAAR1 agonist and 5-HT1A agonist without D2 receptor blockade	Phase 2 RCT (NCT02969382); completed Phase 3 DIAMOND trials	Phase 2 demonstrated significant PANSS improvement versus placebo; subsequent Phase 3 studies yielded mixed results, highlighting challenges in achieving consistent clinical benefit	Generally well tolerated; minimal EPS and metabolic effects
Pimavanserin (adjunctive)	Selective 5-HT2A receptor inverse agonist	Phase 2 ADVANCE (NCT02970305) and Phase 3 ADVANCE-2 (NCT04531982) studies of adjunctive pimavanserin in predominant negative symptoms of schizophrenia; FDA-approved for Parkinson's disease psychosis	Phase 2 studies suggested modest improvement in negative symptoms when used adjunctively with stable antipsychotic treatment; however, subsequent phase 3 findings failed to confirm these benefits.	Well tolerated; minimal motor or metabolic effects
Lumateperone	Multimodal: 5-HT2A antagonism, glutamatergic modulation, and partial dopamine modulation with low D2 occupancy	Phase 3 RCTs in schizophrenia (e.g., NCT01499563)	Significant reduction in PANSS vs placebo in acute schizophrenia	Favorable metabolic profile and low EPS risk
Glycine transporter inhibitors (e.g., bitopertin)	Enhancement of NMDA receptor function via increased synaptic glycine	Phase 2/3 trials in negative symptoms	Mixed efficacy results; initial phase 2 signals not consistently replicated in phase 3	Generally well tolerated
mGluR2/3 agonists (e.g., pomaglumetad)	Metabotropic glutamate receptor 2/3 activation, modulating glutamatergic signaling	Phase 2/3 clinical trials	Early positive signals but phase 3 trials failed to replicate efficacy	Favorable safety but insufficient efficacy

^#^[Based on: 19, 22, 25, 26, 65, 66, 73–80].

AEs, adverse event; BID, twice daily; D2, dopamine D2 receptor; EPS, extrapyramidal symptoms, LS, least squares, mGluR, metabotropic glutamate receptor; NMDA, N-methyl-D-aspartate; PANSS, Positive and Negative Syndrome Scale; RCT, randomized controlled trial; TAAR1, trace amine-associated receptor1.

### 8.1 TAAR1 agonists

TAAR1 is another emerging, non-dopaminergic therapeutic target for the treatment of schizophrenia. TAAR1 is an intracellular G-protein-coupled receptor found in monoaminergic neurons, where it modulates neurotransmitters such as dopamine, serotonin, and glutamate.

Animal studies suggest that TAAR1 activation influences the excitation of dopaminergic neurons in the midbrain and reduces dopamine release in striatal pathways. This mechanism may enable antipsychotic effects without directly blocking D_2_ receptors (81, 82), offering an indirect approach to addressing the dopaminergic dysregulation observed in psychosis.

In a randomized phase 2 trial in patients with acute schizophrenia, ulotaront significantly reduced PANSS total scores compared with placebo while demonstrating minimal extrapyramidal and metabolic adverse effects (73). These encouraging findings led to the phase 3 DIAMOND clinical program. However, subsequent studies yielded mixed results, with inconsistent separation from placebo across different analyses and study populations. Despite these challenges, clinical development has continued, reflecting ongoing interest in TAAR1 agonism as a novel non-dopaminergic approach to schizophrenia treatment (74).

Another compound targeting TAAR1 is ralmitaront (RO6889450). It exhibits lower intrinsic activity and minimal serotonergic activity (compared to ulotaront), but failed to demonstrate clinical efficacy in Phase 2 trials, leading to the discontinuation of its development and underscoring the uncertainty that still surrounds TAAR1-based approaches (83). Despite these setbacks, TAAR1 agonism remains a potentially valuable avenue of research, representing a fundamentally different method for regulating dopaminergic tone without the need for direct receptor blockade (84, 85).

### 8.2 Glutamatergic modulation

The glutamate hypothesis of schizophrenia posits that NMDA receptor hypofunction contributes to the pathophysiology of positive, negative, and cognitive symptoms. This view is based on experimental and clinical observations that NMDA receptor antagonists, such as ketamine and phencyclidine (PCP), can induce schizophrenia-like symptoms, including psychotic symptoms, cognitive deficits, and negative symptoms in healthy individuals (86, 87).

From a neurobiological perspective, reduced NMDA receptor function of inhibitory GABAergic interneurons is thought to cause cortical disinhibition. This, in turn, disrupts dopaminergic signaling pathways, linking the glutamatergic and dopaminergic systems in the pathology of the disease (88, 89). This comprehensive approach has influenced the development of treatments aimed at enhancing the function of NMDA receptors, rather than focusing solely on dopamine receptors.

#### 8.2.1 Glycine-site modulation of NMDA receptors

One of the main strategies has focused on increasing the synaptic availability of co-agonists at the glycine site of the NMDA receptor. Drugs being investigated include glycine transporter-1 (GlyT1) inhibitors (e.g., bitopertin); direct glycine supplementation; and D-serine and other D-amino acids.

Bitopertin showed promise in initial phase 2 trials, particularly in patients with predominant negative symptoms (79). However, subsequent, larger phase 3 trials failed to meet their primary efficacy endpoints, leading to the halt of its clinical development (76).

Similarly, adjunctive D-serine demonstrated benefits in several randomized trials (90, 91), whereas glycine supplementation produced inconsistent effects (92). Subsequent meta-analyses suggested modest overall efficacy of NMDA co-agonist strategies, although considerable heterogeneity remains (93).

#### 8.2.2 Metabotropic glutamate receptor modulation

Other studies have focused on metabotropic glutamate receptors (mGluRs), particularly group II receptors (mGluR2/3), which regulate presynaptic glutamate release.

The mGluR2/3 agonist, pomaglumetad methionil (LY2140023), initially showed promising results in randomized controlled trials, demonstrating antipsychotic activity comparable to olanzapine (94). However, subsequent, larger phase 3 trials failed to demonstrate significant benefit compared to placebo, leading to its discontinuation (80).

These somewhat disappointing results may be due to several factors, including patient heterogeneity, lack of precise stratification, potential compensatory mechanisms in the brain, or insufficient *in vivo* targeting of the drug (72).

#### 8.2.3 Other glutamatergic strategies

Other interventions being investigated include mGluR5 positive allosteric modulators (PAMs), which aim to indirectly improve NMDA receptor function, and AMPA receptor modulators (ampakines), which are intended to enhance synaptic plasticity and cognitive performance (5, 72). Another approach involves sarcosine (N-methylglycine), a GlyT1 inhibitor that increases NMDA receptor co-agonist availability. Preliminary randomized clinical studies suggested that sarcosine adjunctive therapy may improve schizophrenia symptoms, particularly negative symptoms (91). Although early-phase studies have demonstrated some potential benefits for negative and cognitive symptoms, none of these glutamatergic strategies has yet resulted in broadly accepted, regulatory-approved, disease-modifying treatments for schizophrenia (95).

### 8.3 Serotonergic modulation targeting negative symptoms

Another strategy that goes beyond dopaminergic action is selective serotonin receptor modulation, specifically targeting the 5-HT2A receptor. This approach is based on evidence that serotonergic dysregulation may contribute not only to psychotic symptoms but also to negative and affective symptoms in schizophrenia. Based on this, atypical antipsychotics with potent 5-HT2A antagonist activity have been developed, which have a better tolerability profile compared to older FGAs (96).

Pimavanserin, a selective 5-HT2A receptor inverse agonist with negligible affinity for dopamine D2 receptors, was initially approved for the treatment of hallucinations and delusions associated with Parkinson’s disease psychosis (75). Owing to its unique non-dopaminergic mechanism, pimavanserin has attracted interest as a potential adjunctive treatment for predominant negative symptoms in schizophrenia.

In the phase 2 ADVANCE trial, adjunctive pimavanserin added to stable antipsychotic therapy resulted in statistically significant improvements in negative symptoms compared with placebo, as assessed using the Negative Symptom Assessment-16 (NSA-16) scale and other secondary outcome measures (77). Although the observed effect size was modest, these findings were considered clinically meaningful given the persistent unmet need for effective treatments targeting negative symptoms. Importantly, pimavanserin demonstrated a favorable tolerability profile, with minimal extrapyramidal effects, negligible metabolic burden, and no clinically relevant dopaminergic adverse effects, consistent with its receptor selectivity (77).

The encouraging phase 2 findings prompted further evaluation in the larger phase 3 ADVANCE-2 trial. However, this study failed to demonstrate statistically significant improvements in the primary or secondary efficacy endpoints compared with placebo (78). The discrepancy between the phase 2 and phase 3 results underscores the heterogeneity and complexity of negative symptoms in schizophrenia and highlights the challenges associated with their assessment. Moreover, secondary negative symptoms related to depression, medication adverse effects, social withdrawal, or cognitive impairment may confound clinical outcomes and complicate the interpretation of therapeutic effects (78).

### 8.4 Multimodal antipsychotic mechanisms

Lumateperone is an approved multimodal antipsychotic included here as an example of reduced D_2_-dependence rather than a purely non-dopaminergic mechanism, illustrating the broader therapeutic shift already underway within modern antipsychotic development. Although it is not strictly a non-dopaminergic agent, its unique receptor profile reflects increasing efforts to reduce reliance on high-occupancy D_2_ antagonism through more balanced multimodal pharmacological strategies. It acts primarily as a high-affinity 5-HT_2_A receptor antagonist while also exhibiting relatively low D_2_ receptor occupancy and indirect glutamatergic modulation (97). This pharmacological profile may contribute to antipsychotic efficacy while potentially reducing extrapyramidal and metabolic adverse effects compared with traditional high-potency dopamine antagonists (19).

The drug also inhibits the serotonin transporter (SERT), which is another element of its multimodal profile and potentially also affects emotional and cognitive symptoms (for a review, see 98).

Data from randomized, controlled phase 2 and 3 trials reveal that lumateperone significantly reduces the PANSS total score in patients with acute episodes of schizophrenia compared to placebo-indicating that its antipsychotic efficacy is clinically relevant (19). These pivotal trials consistently demonstrate improvements in symptom severity across diverse populations. Lumateperone also has a relatively favorable safety and tolerability profile. Reports highlight a low rate of extrapyramidal symptoms, a modest effect on prolactin, limited metabolic side effects, and minimal risk of weight gain (19). This safety pattern aligns well with its moderate D2 receptor occupancy and balanced action on serotonin receptors. In summary, the drug’s pharmacodynamics reflect a growing trend in schizophrenia treatment: a shift toward multimodal receptor targeting, integrating serotonergic, glutamatergic, and dopaminergic systems, aiming to maintain efficacy without the burden of excessive side effects (98).

These features distinguish lumateperone from many SGAs. Imaging and pharmacodynamic studies suggest that it is not simply about potent dopamine receptor blockade; its balanced modulation appears sufficient to achieve good clinical outcomes. This challenges the notion that high D2 receptor occupancy is necessary for effective antipsychotic treatment. This multimodal strategy aligns with contemporary views that emphasize dysregulation across entire neural networks rather than dysfunction limited to a single neurotransmitter system.

### 8.5 Emerging muscarinic strategies beyond KarXT

While KarXT represents the first clinically successful muscarinic-based antipsychotic, newer compounds aim to refine this therapeutic strategy through greater receptor subtype selectivity and improved tolerability.

Muscarinic positive allosteric modulators (PAMs) offer a more targeted approach to cholinergic signaling in schizophrenia. Unlike direct muscarinic agonists, which frequently produce peripheral cholinergic adverse effects, PAMs enhance receptor responsiveness only in the presence of endogenous acetylcholine. This mechanism may preserve therapeutic efficacy while improving receptor selectivity and reducing off-target effects.

Preclinical studies have consistently demonstrated antipsychotic-like effects of M4 positive allosteric modulators (PAMs), including reversal of amphetamine-induced hyperlocomotion and normalization of dopaminergic activity within striatal circuits (31, 99–101). Among the best-characterized compounds are VU0152100 and VU0467154.

A particularly important next-generation candidate is emraclidine, an M4-preferring PAM. Early clinical studies suggested antipsychotic efficacy with a favorable tolerability profile (102). However, subsequent phase 2 EMPOWER studies failed to meet their primary efficacy endpoints, highlighting the challenges of translating promising mechanistic hypotheses into successful late-stage clinical outcomes (103). Nevertheless, highly selective M4-targeting agents remain valuable tools for clarifying the relative contributions of M1 and M4 receptor signaling to antipsychotic effects and may further advance precision muscarinic therapeutics in schizophrenia.

Collectively, these developments suggest that muscarinic pharmacology may evolve beyond dual M1/M4 agonism toward increasingly selective receptor-targeted interventions with potentially improved efficacy–tolerability profiles.

### 8.6 Lessons from non-dopaminergic drug development and biological heterogeneity of schizophrenia

The development of non-dopaminergic therapies for schizophrenia has been characterized by both innovation and repeated clinical setbacks. Several initially promising compounds, including the TAAR1 agonist ralmitaront, the mGluR2/3 agonist pomaglumetad methionil, and the glycine transporter inhibitor bitopertin, failed to demonstrate sufficient efficacy in later-stage clinical trials despite encouraging early-phase findings (76, 79, 80, 94).

These outcomes underscore the substantial translational challenges that characterize schizophrenia drug development. Potential contributors include inadequate patient enrichment strategies, limited biomarker-guided stratification, high placebo response rates, and clinical endpoints that may insufficiently capture mechanism-specific therapeutic effects. Importantly, negative trial outcomes do not necessarily invalidate the underlying biological hypotheses. Rather, they may reflect limitations in matching targeted interventions to the patient populations most likely to benefit from them.

Growing evidence suggests that schizophrenia encompasses considerable biological heterogeneity, with multiple neurobiological pathways potentially contributing to similar clinical manifestations. Consequently, treatments targeting distinct neurotransmitter systems may demonstrate efficacy only within specific patient subgroups rather than across the entire diagnostic spectrum.

### 8.7 Comparative perspective

Taken together, these observations suggest that future progress in schizophrenia therapeutics may depend not only on the identification of novel molecular targets but also on improved biological stratification of patients and more precision-oriented clinical trial design.Collectively, the therapeutic approaches reviewed in this chapter illustrate the growing diversification of schizophrenia drug development beyond traditional dopamine D_2_ receptor antagonism. Although many non-dopaminergic programs have produced mixed or inconclusive clinical results, they have substantially expanded understanding of the neurobiological mechanisms underlying psychosis and highlighted several potentially actionable therapeutic pathways.

Together, these developments suggest that schizophrenia treatment is gradually evolving from a predominantly dopamine-centered model toward a broader framework incorporating multiple neurobiological mechanisms and therapeutic targets.

## 9 Implications for negative and cognitive symptoms

Negative and cognitive symptoms remain among the most challenging issues in the treatment of schizophrenia. These symptoms are closely associated with difficulties in daily life, such as impaired functioning, unemployment, and reduced quality of life.

Traditional antipsychotic medications, which primarily target dopamine receptors, generally demonstrate poor results in these areas. Meta-analyses indicate that any improvement in negative symptoms observed with these medications is often secondary to improvements in positive symptoms rather than reflecting a direct effect on the negative symptoms themselves (21).

Activation of muscarinic receptors represents another pathway that may more directly influence brain circuits involved in cognition and motivation.

M1 receptors are highly expressed in the prefrontal cortex and hippocampus, regions important for cognitive function. They play a role in synaptic plasticity, neuronal excitability, and NMDA receptor signaling. Stimulation of M1 receptors enhances long-term potentiation, which is essential for learning and memory (104).

Preclinical studies provide additional support for this view. For example, xanomeline can reverse deficits in latent inhibition, a behavioral marker relevant to schizophrenia, induced by compounds such as amphetamine, MK-801, or scopolamine (54).

Neuroimaging evidence supports this view, showing that muscarinic receptor agonists can restore disrupted large-scale neural network connectivity. In rodent models, xanomeline was able to reverse changes in functional connectivity induced by NMDA receptor antagonists, which may be related to cognitive processing in schizophrenia (105).

Clinical data on these cognitive effects are still in the early and exploratory phase. Current evidence for cognitive benefit remains exploratory and derives primarily from *post hoc* analyses rather than prospectively designed cognition trials. Some analyses from the EMERGENT trials indicate benefits in specific cognitive domains in patients who were impaired at baseline, but dedicated cognitive testing will be necessary to draw more definitive conclusions (27). Future studies should incorporate standardized cognitive test batteries, such as the MATRICS Consensus Cognitive Battery (MCCB), to better understand how therapies targeting muscarinic receptors may impact cognitive function.

## 10 Current clinical position of KarXT

The development of KarXT (xanomeline–trospium) provides the clearest evidence to date that effective antipsychotic treatment can be achieved without direct dopamine D_2_ receptor antagonism (22, 24–26, 67, 69). Compared with other non-dopaminergic approaches, including TAAR1 agonists, serotonergic modulators, and glutamatergic agents, KarXT currently possesses the most robust and reproducible efficacy data, supported by replicated phase 3 clinical trials and long-term extension studies.

The available evidence indicates that KarXT produces clinically meaningful improvements in positive symptoms while maintaining a favorable extrapyramidal and metabolic safety profile. Although potential benefits for cognitive and negative symptoms have been suggested, these findings remain exploratory and require confirmation in dedicated prospective studies.

Despite these advances, several important questions remain unresolved. Active-comparator studies directly comparing KarXT with established second-generation antipsychotics are currently lacking, limiting conclusions regarding comparative efficacy, tolerability, and treatment persistence. Similarly, evidence regarding relapse prevention, treatment-resistant schizophrenia, long-term functional recovery, and real-world effectiveness remains limited.

At present, KarXT should therefore be viewed as an important addition to the antipsychotic armamentarium rather than a definitive replacement for existing treatment strategies. Ongoing clinical experience and future comparative studies will be essential for determining its optimal position within schizophrenia treatment algorithms.

### 10.1 Toward precision muscarinic therapeutics in schizophrenia

One of the most important future challenges will be integrating muscarinic-based therapies into the broader framework of precision psychiatry. Increasing evidence suggests that schizophrenia is not a single biologically uniform disorder but rather a heterogeneous group of syndromes involving partially distinct neurobiological mechanisms. This heterogeneity raises the possibility that different therapeutic mechanisms, including muscarinic, TAAR1-mediated, glutamatergic, and serotonergic approaches may prove most effective in specific biological subgroups of patients rather than across the entire diagnostic spectrum.

Recent real-world observations support this possibility. Early adjunctive use of Cobenfy has been associated with differential response patterns, with prominent negative symptoms and stimulant use history emerging as potential predictors of benefit (106). Such findings remain preliminary but highlight the need for biologically and clinically informed patient stratification.

Additional support for this concept comes from postmortem and neuroimaging studies suggesting that approximately 25–30% of patients with schizophrenia demonstrate reduced cortical M1 receptor expression, sometimes referred to as a muscarinic receptor-deficient subgroup (29, 30). Importantly, this observation should not be interpreted as evidence that schizophrenia is primarily a cholinergic disorder. Rather, it illustrates the biological heterogeneity of the illness and supports the possibility that muscarinic therapies may exhibit differential efficacy across patient populations.

Future progress toward precision muscarinic therapeutics will likely require the identification of clinically useful biomarkers capable of defining biologically relevant patient subgroups. Candidate approaches include PET-based receptor imaging, transcriptomic and gene-expression signatures, electrophysiological measures of cortical network dysfunction, cognitive phenotyping, and inflammatory or neurodevelopmental biomarkers. Although none are currently validated for routine clinical use, such tools may eventually facilitate mechanism-based treatment selection and improve clinical trial enrichment strategies.

Combination therapies also represent a promising future direction. Muscarinic agents may ultimately be combined with therapies targeting complementary neurotransmitter systems, potentially allowing broader symptom control while minimizing adverse effects associated with high levels of dopamine receptor blockade. Nevertheless, KarXT has already provided a major proof of concept that effective antipsychotic treatment can be achieved through non-dopaminergic mechanisms and has opened a new avenue for future therapeutic development.

## 11 Conclusions

The advent of KarXT represents one of the most important advances in antipsychotic drug development in recent years. By selectively activating central muscarinic M1 and M4 receptors without blocking dopamine D_2_ receptors, KarXT demonstrates that influencing other neurotransmitter systems can provide effective antipsychotic action. KarXT demonstrates robust efficacy for positive symptoms, while possible effects on negative and cognitive domains remain preliminary and require dedicated prospective evaluation. Compared to other new drugs not targeting D_2_ receptors, such as TAAR1 agonists or glutamatergic agents, KarXT currently has the strongest clinical validation, based on replicated Phase 3 studies and supported by long-term extension studies.

These findings support a multidimensional understanding of schizophrenia, in which cholinergic dysfunction may represent one clinically actionable pathway among several interacting neurobiological mechanisms. Future research will determine how muscarinic therapies can be optimally integrated into biologically informed and mechanism-based treatment strategies for schizophrenia.

In summary, there is the potential to move toward a pharmacotherapy for schizophrenia that is based on treatment mechanisms and tailored to individual needs, offering treatment approaches that go beyond the traditional dopamine-focused paradigm. Rather than redefining schizophrenia as a cholinergic disorder, the emergence of KarXT reinforces the need to move toward biologically informed, mechanism-based, and subtype-sensitive treatment models. The identification of muscarinic receptor-deficient subgroups further supports a stratified treatment framework, emphasizing patient selection rather than disease redefinition. Whether muscarinic modulation ultimately represents a broadly applicable antipsychotic strategy or a mechanism preferentially relevant to biologically defined patient subgroups remains an important open question for future research.
